# *Notes from the Field:* Investigation of an Outbreak of *Salmonella* Paratyphi B Variant L(+) tartrate + (Java) Associated with Ball Python Exposure — United States, 2017

**DOI:** 10.15585/mmwr.mm6719a7

**Published:** 2018-05-18

**Authors:** Vikram Krishnasamy, Lauren Stevenson, Lia Koski, Marilee Kellis, Betsy Schroeder, Madhura Sundararajan, Stephen Ladd-Wilson, Ashley Sampsel, Mike Mannell, Andrew Classon, Darlene Wagner, Kelley Hise, Heather Carleton, Eija Trees, Linda Schlater, Kristina Lantz, Megin Nichols

**Affiliations:** ^1^Epidemic Intelligence Service, CDC; ^2^Division of Foodborne, Waterborne, and Environmental Diseases, National Center for Emerging and Zoonotic Infectious Diseases, CDC; ^3^CAITTA, Inc., Herndon, Virginia; ^4^Oak Ridge Institute for Science and Education, Oak Ridge Tennessee; ^5^Arizona Department of Health Services; ^6^Indiana State Department of Health; ^7^Oregon Health Authority, Public Health Division; ^8^Oklahoma State Department of Health; ^9^IHRC, Inc., Atlanta, Georgia; ^10^National Veterinary Services Laboratories, Science, Technology and Analysis Services, Veterinary Services, Animal and Plant Health Inspection Service, U.S. Department of Agriculture, Washington, DC.

In July 2017, PulseNet, the national molecular subtyping network for foodborne disease surveillance, identified a cluster of five *Salmonella* Paratyphi B variant L(+) tartrate + (Java) clinical isolates that were indistinguishable by pulsed-field gel electrophoresis (PFGE). Initial questionnaires administered by state and local health department investigators indicated animal exposure as a possible source of infection, with all five patients reporting snake exposure. An outbreak investigation was initiated to identify the source of infection.

A case was defined as isolation of *Salmonella* Paratyphi B variant L(+) tartrate + (Java) from June 17, 2017, to July 23, 2017, with a PFGE enzyme pattern indistinguishable from the outbreak strain. A snake-specific questionnaire regarding snake type, snake purchase location, and reptile food, including feeder rodents, was developed and administered to patients by state and local health department investigators. In addition, animal and environmental sampling was conducted at patient residences. Traceback of patients’ snakes was conducted by contacting snake purchase locations to identify common suppliers. Finally, whole genome sequencing (WGS) was performed on clinical, environmental, animal, and pet food isolates to further characterize their genetic relatedness, measured in single nucleotide polymorphism (SNP) differences (*1*).

Five cases were identified in four states: one each in Arizona, Oklahoma, and Oregon, and two in Indiana from different households with no epidemiologic link. Median patient age was 10 years (range = <1–40 years), and four were female. No patient was hospitalized, and no deaths occurred. Five patients or their proxies completed the snake-specific questionnaire, four of whom reported exposure to a ball python in the residence. Ball python sampling occurred in the Arizona, Oregon, and one of the Indiana patient residences by sampling the python cloaca, environment, water, and bedding. Feeder rodent sampling occurred in the Arizona and Indiana patient residences. No common suppliers of either ball pythons or feeder rodents were identified by traceback.

A ball python bedding sample from the Arizona residence and a ball python cloacal sample from the Indiana residence yielded *Salmonella* Paratyphi B variant L(+) tartrate + (Java). Sampling also identified *Salmonella* Mbandaka from a feeder rodent and a ball python at the Arizona residence. In addition, Oregon ball python environmental samples yielded *Salmonella* Oranienburg. Further investigation of the *Salmonella* Mbandaka and Oranienburg isolates did not identify human illnesses linked to snake exposure. Finally, the National Veterinary Services Laboratories (NVSL) identified three *Salmonella* Paratyphi B var L(+) tartrate + (Java) isolates from other pythons in 2017.

WGS analysis indicated that among human isolates, only the two Indiana patient isolates were closely related genetically (0–2 SNP differences) (Figure). The Arizona, Oklahoma, and Oregon patient isolates were not closely related genetically to each other or to the Indiana patient isolates. In addition, the Arizona ball python bedding sample was indistinguishable from the Arizona patient isolate (0 SNP differences), and the Indiana ball python and environmental samples were closely related to both Indiana patient isolates (0–2 SNP differences). Finally, the three python isolates identified at NVSL were not closely related to any human, animal, or environmental isolate.

**FIGURE:**
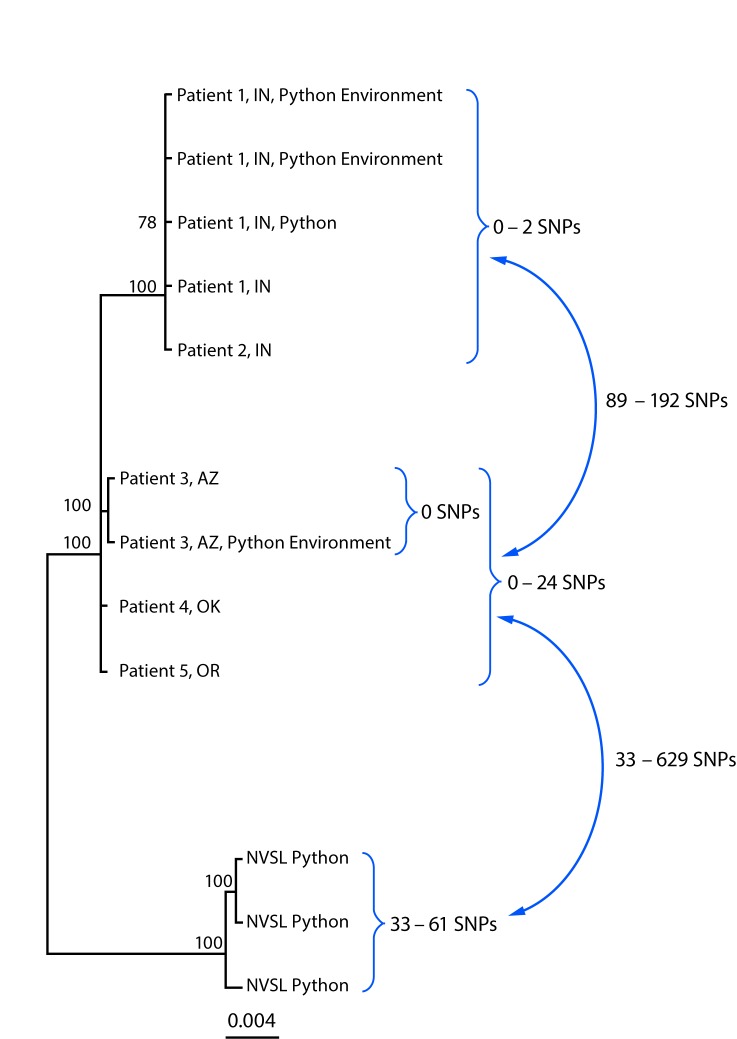
Whole-genome sequencing analysis of isolate genomes of *Salmonella* Paratyphi B variant L(+) tartrate + (Java) from human, from *Python regius,* and from environmental sources associated with the outbreak investigation **—** United States, 2017* **Abbreviations:** AZ = Arizona; hqSNP = high-quality single nucleotide polymorphism; IN = Indiana; NVSL = National Veterinary Services Laboratories; OK = Oklahoma; SNPs = single nucleotide polymorphisms. * Analysis performed using Lyve-SET version 1.1.4f (https://github.com/lskatz/lyve-SET) and hqSNPs were called at >20x coverage, >95% read support, and allowed Flanking set to five base pairs. Reference used was draft assembly of Arizona case (78 contigs) without phage masking. ^†^ Three isolates from different pythons submitted to NVSL for testing.

*Python regius*, also known as ball or royal pythons, are a python species native to sub-Saharan Africa. Their tame nature and small size relative to other pythons make them popular pets in the United States (*2*,*3*). However, like other reptiles, ball pythons are known carriers of multiple *Salmonella* serovars (*4*). As a result, CDC recommends that children aged <5 years avoid contact with reptiles (*5*). The median age of patients in this investigation was 10 years, indicating that children aged >5 years are also at risk for illness. Identifying and investigating zoonotic clusters of salmonellosis require a One Health (*6*) approach using multidisciplinary collaboration with departments of agriculture and health.
